# Feature integration of [^18^F]FDG PET brain imaging using deep learning for sensitive cognitive decline detection

**DOI:** 10.1371/journal.pone.0341995

**Published:** 2026-07-21

**Authors:** Youjin Lee, Seonguk Kim, Sangil Kim, Yeona Kang, for the Alzheimer’s Disease Neuroimaging Initiative

**Affiliations:** 1 Department of Mathematics, Pusan National University, Busan, Republic of Korea; 2 Department of Mathematics, Defiance College, Defiance, Ohio, United States of America; 3 Department of Mathematics, Howard University, Washington, United States of America; IRCCS Foundation Stella Maris: IRCCS Fondazione Stella Maris, ITALY

## Abstract

**Background:**

Distinguishing individuals with cognitive decline (CD), including early Alzheimer’s disease, from cognitively normal (CN) individuals is essential for improving diagnostic accuracy and enabling timely intervention. Positron emission tomography (PET) captures metabolic brain alterations associated with CD, but its broader application is often limited by cost and radiation exposure. To enhance the clinical utility of PET while addressing data limitations, we propose a data-efficient framework that integrates complementary multi-scale PET representations at voxel-level and region-level.

**Methods:**

Voxel-level features were extracted using convolutional neural networks (CNN) or principal component analysis networks (PCANet) from [¹⁸F]FDG PET imaging. Region-level features were derived from standardized uptake value ratio measurements across predefined brain regions and processed using a deep neural network (DNN). These voxel- and region-level information are integrated through direct concatenation. For the final prediction, different machine learning models and ensemble technique were applied. The models were trained and validated using 5-fold cross-validation on PET scans from 252 participants in the Alzheimer’s Disease Neuroimaging Initiative, comprising 118 CN and 134 CD subjects. Additional correlation analysis and disease classification comparison with the Mini-Mental State Examination (MMSE) were also performed.

**Results:**

In 5-fold cross-validation, CNN, PCANet, and DNN models achieved classification accuracies of 0.69 ± 0.04, 0.69 ± 0.06, and 0.82 ± 0.06, respectively. The integrated DNN-CNN model using direct concatenation yielded the highest accuracy (0.87 ± 0.05), with a 6.33% improvement in accuracy and reduced standard deviation relative to the DNN-only model. Overall, there were an increase of 14.22% in Recall (0.77 to 0.88) and an increase of 7.92% in F1-Score (0.82 to 0.88). Moreover, the predicted probability of CD showed a significant correlation with MMSE scores, and the model achieved higher accuracy, recall, and F1-score than MMSE-based classification.

**Conclusion:**

Combining complementary voxel-level and region-level PET representations with deep learning improved classification performance over single-representation models, particularly by enhancing sensitivity to cognitive decline. These findings support the potential utility of multi-scale FDG-PET representations for machine learning-based cognitive decline detection.

## Introduction

Alzheimer's disease (AD) is a progressive neurodegenerative disorder that severely impairs cognitive function and activities of daily living (ADLs) [1–3]. As of March 2023, the World Health Organization estimates that over 55 million people worldwide are living with dementia, with AD being the most common form of dementia accounting for 60–70% of cases [4]. AD primarily affects brain regions responsible for memory, language, and reasoning, leading to a gradual decline independence and quality of life [1–3]. According to the Centers for Disease Control and Prevention, symptoms typically emerge after the age of 60, with the risk of AD doubles approximately every five years beyond age 65 [5,6].

Mild cognitive impairment (MCI), often regarded as a precursor to AD [7,8], involves noticeable cognitive decline that does not yet interfere significantly with ADLs [3]. However, over 50% of individuals with MCI progress to dementia within five years, with AD being the predominate cause [8,9]. Evidences suggests that early intervention at the MCI stage may slow or even reverse the disease progression [3,10]. Therefore, MCI related to AD represents a critical window for early diagnosis and treatment [11–15]. For this reason, AD and MCI are often grouped under the broader term “cognitively decline” (CD), highlighting the importance of early and reliable identification in both research and clinical setting.

Among current diagnostic tools, positron emission tomography (PET) plays an important role in the evaluation of AD, with different tracers providing complementary diagnostic information [16–18]. [^18^F]fluorodeoxyglucose (FDG) PET enables the evaluation of cerebral glucose metabolism, revealing underlying neurodegenerative processes [19]. The standardized uptake value ratio (SUVr) has been widely adopted as a semi-quantification metric in both research and clinical practice due to its relative simplicity and ease of implementation [20,21].

Recent advances in deep learning have expanded the application of medical imaging for automated disease classification, including brain tumor segmentation using magnetic resonance imaging (MRI) [22], coronavirus disease 2019 detection from lung computed tomography scans [23], and AD classification (MRI and PET) [24]. Studies have explored diverse approaches to AD classification using multiple imaging modalities (e.g., MRI, functional MRI, PET) and varying input levels (e.g., 3D ROI-based, 3D subject-level, 3D patch-level, and 2D slice-level data) [25,26]. Hybrid models combining traditional machine learning (ML) with deep learning methods, as well as multi-institutional collaborations, have also been reported [26,27].

For instance, a study using 2,552 PET scans from 836 participants conducted One-vs-All binary classification to distinguish each diagnostic group from the others, reporting classification accuracies of 0.74 (CN), 0.59 (MCI), and 0.78 (AD) [28]. Another study evaluated a model on 822 subjects (472 AD, 350 MCI) and achieved 0.79 accuracy on a hold-out test set (10% of the dataset) and 0.80 balanced accuracy during external validation [29]. Despite relatively limited datasets, these results highlight the growing feasibility of PET-based diagnostics in data-limited settings.

PET brain image offers a distinct advantage in detecting physiological changes earlier than structural imaging, offering valuable insights for diagnosis and disease monitoring [30,31]. However, PET imaging requires radioactive tracers and dedicated imaging infrastructure, making data acquisition expensive and comparatively limited relative to other imaging modalities [32,33]. Consequently, developing models that can achieve robust performance with limited training data may enhance the clinical utility of PET-based diagnostic tools.

In our study, we present a deep learning framework to identify CD using a limited number of FDG PET scans. Our approach integrates both voxel-level and region-level features from the same FDG PET modality, enabling more comprehensive analysis. We validated the performance of our feature fusion strategy across different dimensional levels and ML classifiers using data from the Alzheimer's Disease Neuroimaging Initiative (ADNI). Finally, to provide insights into the model interpretability, we examined the relationship between model-predicted probabilities of CD and the clinical metrics, and compared their disease classification performance.

## Materials and methods

### Data collection and characteristics

Data used in the preparation of this article were obtained from the Alzheimer’s Disease Neuroimaging Initiative (ADNI) database (adni.loni.usc.edu). The ADNI was launched in 2003 as a public-private partnership, led by Principal Investigator Michael W. Weiner, MD. The primary goal of ADNI has been to test whether serial MRI, PET, other biological markers, and clinical and neuropsychological assessment can be combined to measure the progression of MCI and early AD.

The research groups included subjects classified as CN, MCI, and AD. A total of 252 subjects were included in the study, consisting of 118 CN individuals and 134 patients, which included 70 individuals with MCI and 64 with AD. Moreover, one PET and one MRI scan were collected for each subject. Additional demographic and clinical variables, including sex, age, and the Mini-Mental State Examination (MMSE), were obtained when available.

The basic characteristic information of the dataset is stated in Table 1. Next, to avoid potential bias in the classification task, the distribution of key demographic variables, specifically sex and age, were examined across the dataset using statistical analysis. Moreover, to facilitate the clinical interpretation of our predictions, we analyzed whether there were significant differences in the distribution of MMSE between groups.

**Table 1:** Basic characteristic information of the dataset.

Characteristic	Total	CN	CD
Total Subjects	252 (100.0%)	118(46.8%)	134(53.2%)
Age, years	75.5 ± 6.4	76.1 ± 5.6	75.1 ± 7.0
Gender (M/F)	132 / 120(52.4% / 47.6%)	61 / 57(51.7% / 48.3%)	71 / 63(53.0% / 47.0%)
MMSE score	26.3 ± 4.9(N = 147)	29.2 ± 1.0(N = 76)	23.2 ± 5.5(N = 71)

The data are presented as mean ± SD or as n (%).

For PET imaging, we selected FDG PET scans. According to the PET protocols [34], the FDG tracer is administered at a dose of 185 MBq (5.0 mCi) with an allowable variation of ±10% across different studies (ADNI1, ADNI GO, ADNI2, ADNI3). The selected data are preprocessed including coregistered average, standard images, and voxel sizes with uniform resolution (described as “Coreg, Avg, Std Img and Vox Siz, Uniform Resolution”). During the preprocessing, cerebellar gray matter is used as the reference region for SUVr normalization, as described in the ADNI PET document [35]. Thus, the collected PET imaging is an SUVr map, representing the relative FDG uptake compared to reference region.

For MRI imaging, T1-weighted images were collected within one month before or after the corresponding PET scan for the subject. This selection ensured that the MRI data was temporally aligned with the PET scans and MRI imaging is to conduct segmentation for region-based PET measurement.

### Multi-representational deep learning framework

**Fig 1:**
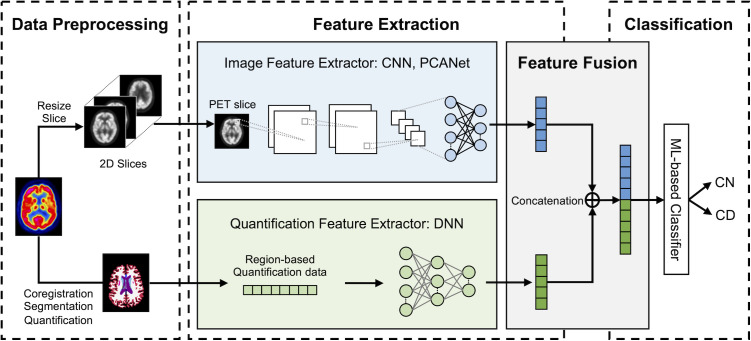
The framework of proposed multi-representational deep learning model.

The framework of our proposed multi-representational deep learning is shown in Fig 1. It consists of four phases: Data Preprocessing, Feature Extraction, Feature Fusion, and Classification. From a single imaging modality—FDG PET—two types of feature representations are derived during preprocessing: 2D PET slices and regional SUVr values. In the deep feature extraction stage, PET images and regional SUVr data are processed using separate deep learning architectures, each specialized for spatial or structured input respectively. The extracted features are integrated via direct concatenation at the feature fusion stage. The resulting fused feature vector is subsequently utilized as the input to the final ML-based classifier with ensemble technique for final prediction

### Data preprocessing

The collected PET images were also utilized as inputs for deep learning models. Initially, the scans were resized to 128 × 128 × 64. To reduce the model size and the number of parameters required, the original 3D PET scans were sliced into 2D images. Specifically, 10 slices were extracted from the central region of the 3D PET scan, focusing on the ventricles, particularly in axial slices, for augmentation purpose during training (training and validation set). Additionally, only one middle slice was obtained for test set.

For region-based analysis, segmentation, coregistration and regional SUVr extraction were conducted. Freesurfer [36] was used for the segmentation of each MRI image. The segmentation output (aseg.mgz) divides the brain into 41 distinct regions, with detailed region label provided in S1 Table. Each PET image was coregistered to the corresponding MRI image, and average regional activity (expressed as SUVr values) was extracted using the segmented masks. Co-registration and SUVr extraction were performed using PMOD (version 3.8; PMOD Technologies Ltd [37]). Regional SUVr values were collected for all subjects and organized into tabular data.

To evaluate the model while ensuring its generalizability and robustness, we conducted 5-fold cross-validation. In each fold, the dataset was split into training, validation, and test sets in a ratio of 64%, 16%, and 20%, respectively. The split was conducted in subject level (the same as PET imaging level) and augmentation is applied after the split. Also, the same set division applied for PET imaging and SUVr values.

### Feature extraction

To integrate two different types of data, regional SUVr (tabular data) and 2D slice image (imaging data), deep learning models are employed to independently extract features from each data type. Deep Neural Networks (DNNs) were employed for tabular data, while Convolutional Neural Networks (CNNs) were utilized for imaging data due to their respective architectures and specialized capabilities, which align with the characteristics of these data types. To address the limitation of a small dataset, we utilized EfficientNetB0 [38], a compact and efficient CNN pretrained on ImageNet, and applied fine-tuning by unfreezing the last four layers to adapt the model to the target task. All detailed architecture of DNN and CNN is stated in S2 and S3 Tables.

Although the performance of CNNs is well recognized in vision tasks including various medical imaging tasks [39], it is also well known that a large size of dataset is required for high accuracy. As aiming to performance on smaller dataset, we additionally employed a simple neural network, Principal Component Analysis (PCA) Network (PCANet) [40]. PCANet follows similar stages as CNN; however, unlike CNNs, it does not require updating the weights of the filters. Instead, the filters are derived from PCA, eliminating the need for gradient-based optimization. This makes PCANet easier to implement and less computationally intensive, particularly for tasks with limited labeled data. All detailed setting for PCANet is stated in S4 Table.

Specifically, the regional-based features were derived from three distinct DNN layers: DNN1 (with a feature dimensionality of 16), DNN2 (32), and DNN3 (64). Additionally, the original tabular data was incorporated into the fusion process and referred to as DNN0 (41). For PET imaging, both a convolutional neural network (CNN) and PCANet were employed. CNN features were extracted from three layers: CNN1 (with a dimensionality of 16), CNN2 (32), and CNN3 (1280). CNN3 corresponds to the final feature representation obtained from the pretrained EfficientNet-B0 model. In the case of PCANet, the extracted feature vector had a dimensionality of 128.

### Feature fusion

The extracted features are subsequently fused in a concatenation manner. There are four feature dimension levels for each PET imaging and regional SUVr data as described above and fusion is implemented in all possible 16 combination ways. Through concatenation, these features form a unified representation, enabling the model to leverage the complementary information contained within both PET imaging and regional SUVr data.

### Classification

ML based classifiers were used to make the final prediction. A total of 12 machine learning classifiers were employed in this study, including Random Forest (RF), Extra Trees (ET), Multi-Layer Perceptron (MLP), Naive Bayes (NB), Gradient Boosting (GB), Logistic Regression (LR), k-Nearest Neighbors (KNN), Support Vector Machine (SVM), and Decision Tree (DT), all implemented using the Scikit-learn library [41]. In addition, eXtreme Gradient Boosting (XGBoost) [42], Light Gradient Boosting Machine (LightGBM) [43], and Categorical Boosting (CatBoost) [44] were utilized via their respective official Python libraries. To further boost model performance, to enhance robustness, we implemented ensemble methods by combining heterogeneous classifiers from different algorithmic families, including RF (tree-based), MLP (neural network), CB (gradient boosting), SVM (kernel-based), and LR (linear model). Ensemble strategies such as hard voting, soft voting, and stacking were applied using the predicted probabilities from each model.

### Model assessment

Our proposed frameworks and baseline models were compared using evaluation metrics such as precision, recall, F1 score, accuracy, and the area under the ROC curve (AUC). All performance metrics were evaluated using 5-fold cross-validation and reported as the mean and standard deviation across folds. For confusion matrix analysis, predictions from all five folds were aggregated to allow for a more stable comparison across models. Among various evaluation metrics, we selected the F1-score as the primary performance indicator, as it provides a balanced assessment of precision and recall as shown in Eq (1), which serves as a more informative metric in clinical classification tasks due to its sensitivity to both false positives (FP) and false negatives (FN) beyond simply considering true positives (TP) and true negatives (TN) [45].


$$\begin{array}{c} \begin{array}{c} \text{F1}=\text{2}\frac{\text{precision}\times \text{recall}}{\text{precision}+\text{recall}}=\frac{\text{2}\times \text{TP}}{\text{2}\times \text{TP}+\text{FP}+\text{FN}} \end{array} \end{array}$$ (1)

### Interpretability analysis

Beyond binary classification, we clinically evaluate our predictions. Each model generates an output as a probability between 0 and 1, which is converted to 0 or 1 using a threshold of 0.5 for final prediction. We assume that the predicted probability may reflect the severity of CD. To examine this, we calculate the Pearson correlation coefficient (r) with MMSE, which is one of well-known cognitive assessment tool for evaluating various cognitive disorders [46].

Furthermore, we compared the discriminative ability between the models and MMSE. To accomplish this, we re-evaluated model performance using only the patients for whom clinical data were available (n = 147). Here, to classify individuals using MMSE, we categorized those with scores less than 24 as CD and those with scores greater than or equal to 24 as CN [47]. For this analysis, we used the same predictions generated during the original 5-fold cross-validation and computed the performance metrics using all available predictions.

## Results

### Data characteristics analysis

The distribution of age for both CN and CD can be considered normally distributed since p-value from the Shapiro-Wilk normality test is greater than 0.05. With the assumption of normality, we conducted t-test and it showed no significant difference in the age distribution between CN and CD (p-value = 0.20). By chi-square test for sex distribution, we observed that there is no significant difference in the distribution of sex between CN and CD (p-value = 0.94). Thus, our dataset constructed for classification task dose not exhibit any bias with respect to sex or age.

Total 147 subjects out of 252 subjects are available for MMSE analysis. Firstly, we observed non-normality in the distribution of MMSE in both groups from the Shapiro-Wilk normality test (p < 0.001). So, the Mann-Whitney U test was employed as non-parametric alternative. According to the Mann-Whitney U test with a one-sided alternative hypothesis (greater), CN had significantly higher MMSE score than CD (p < 0.001). As a well-established clinical indicator, MMSE showed a statistically significant difference and is thus considered a representative clinical metric for further analysis.

### Baseline models performance

Firstly, the performance of baseline model is examined. DNN is employed for regional SVUr data (tabular data) while CNN and PCANet are utilized for PET imaging. Since PCANet is only a feature extractor, 12 ML classifiers are employed to make the final prediction (S5 Table). Among them, XGB shows the highest average F1-score - the primary evaluation metric – and showed superior results across all other metrics except for recall. Table 2 presents the performance of the three baseline models. Among baseline models, DNN achieved the best performance across all metrics, with an average F1-score of 0.82. However, CNN, like DNN, achieves higher precision, while PCANet shows a substantially higher recall.

**Table 2:** Comparison of baseline model – DNN, CNN, PCANet with XGB.

Data Type	Model	Accuracy	Precision	Recall	F1-Score	AUC
Regional SUVr	DNN	**0.82 ± 0.06**	**0.89 ± 0.08**	**0.77 ± 0.12**	**0.82 ± 0.08**	**0.89 ± 0.03**
PET imaging	CNN	0.69 ± 0.04	0.78 ± 0.07	0.59 ± 0.04	0.67 ± 0.04	0.75 ± 0.05
	PCANet with XGB	0.69 ± 0.06	0.69 ± 0.05	0.75 ± 0.08	0.72 ± 0.05	0.69 ± 0.06

Bold text: the highest average value for each metric

### Fusion model performance comparison

To investigate the performance models across various combinations of feature levels and final classifiers, we firstly fixed classifier to RF, which shows the most frequently best-performing classifier, and varied the feature levels. Overall, DNN2 has higher average accuracy of 0.86 with standard deviation 0.01 (DNN0: 0.81 ± 0.02, DNN1: 0.85 ± 0.01, DNN3: 0.084 ± 0.01) for regional SUVr data. In the case of PET imaging, CNN3 has slightly higher average accuracy of 0.85 with standard deviation 0.02 (PCANet: 0.84 ± 0.01, CNN1: 0.84 ± 0.02, CNN3: 0.83 ± 0.03) as shown in Table 3. The highest F1-score shows 0.87 with the combination of (DNN2, CNN1) and (DNN2, CNN2) (Table 3). They also outperformed in other metrics excepts, and the evaluation results with all metrics for all combinations are reported in S6 Table.

**Table 3:** Average F1-score of each feature levels with RF.

	DNN0	DNN1	DNN2	DNN3	AVG	SD
**PCANet**	0.84	0.84	0.85	0.84	0.84	0.01
**CNN1**	0.81	0.85	**0.87**	0.84	0.84	0.02
**CNN2**	0.82	0.86	**0.87**	0.85	**0.85**	0.02
**CNN3**	0.78	0.85	0.86	0.82	0.83	0.03
**AVG**	0.81	0.85	**0.86**	0.84		
**SD**	0.02	0.01	0.01	0.01		

(DNN# and CNN# show different layer types)

Next, fixing the latent variable dimension for (DNN2, CNN1), we conducted a comparative analysis of all ten ML classifiers. Also, ensemble approaches using 5 ML models (RF, MLP, CB, SVM, and LR), denoted by Ensemble, were applied with soft voting, stacking, and hard voting. As shown in S7 Table, RF outperforms other individual classifiers in all evaluation metrics including a F1-score of 0.87 among single ML classifiers. While the ensemble approach yields marginally increasing in all metrics: Accuracy (0.86 to 0.87), Precision (0.88 to 0.89), Recall (0.86 to 0.88), F1-scores (0.87 to 0.88) and AUC (0.86 to 0.88) (S8 Table).

### Class-wise performance analysis

All baseline and fusion, ensemble models are summarized in Table 4. We denoted the fusion model using RF with features from DNN2 and CNN1 as Fusion 1 and the combination of DNN2 and CNN2 as Fusion 2. Corresponding best ensemble models are referred to as Ensemble 1 and Ensemble 2.

**Table 4:** Summary of baseline, fusion, and ensemble models’ performance.

Model	Feature	Accuracy	Precision	Recall	F1-Score	AUC
DNN	–	0.82 ± 0.06	0.89 ± 0.08	0.77 ± 0.12	0.82 ± 0.08	**0.89 ± 0.03**
CNN	–	0.69 ± 0.04	0.78 ± 0.07	0.59 ± 0.04	0.67 ± 0.04	0.75 ± 0.05
PCANet	–	0.69 ± 0.06	0.69 ± 0.05	0.75 ± 0.08	0.72 ± 0.05	0.69 ± 0.06
Fusion 1	DNN2|CNN1	0.86 ± 0.05	0.88 ± 0.07	0.86 ± 0.03	0.87 ± 0.04	0.86 ± 0.05
Fusion 2	DNN2|CNN2	0.86 ± 0.06	**0.89 ± 0.09**	0.86 ± 0.03	0.87 ± 0.05	0.86 ± 0.06
Ensemble 1	DNN2|CNN1	**0.87 ± 0.05**	**0.89 ± 0.06**	**0.88 ± 0.04**	**0.88 ± 0.05**	0.88 ± 0.04
Ensemble 2	DNN2|CNN2	**0.87 ± 0.05**	0.88 ± 0.07	**0.88 ± 0.04**	**0.88 ± 0.05**	0.87 ± 0.05

Compared to DNN – the best performing baseline model, both fusion and ensemble models show improved performance. Overall, the average performance become better with smaller standard deviation as a result of feature integration. With precision maintained at a similar level, the substantial improvement in recall indicates that the model has become more sensitive to true positive (CD) detection.

**Fig 2:**
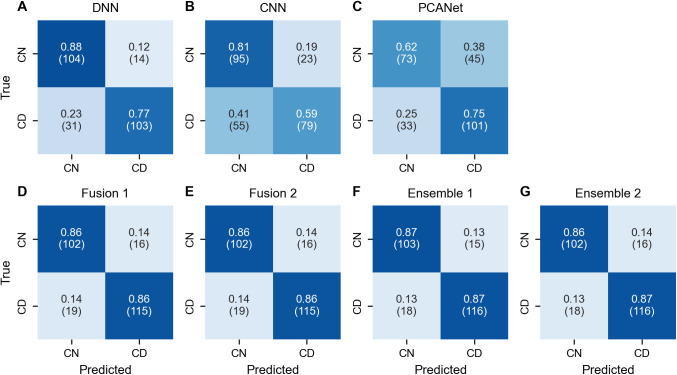
Confusion matrix for baseline, fusion, and ensemble models.

According to the aggregate confusion matrix in Fig 2, both DNN and CNN models demonstrate strong performance in predicting true negatives (CN), with accuracy of 0.88 and 0.81, respectively, whereas PCANet shows a lower prediction in this regard. Although PCANet shows the higher accuracy in true positives (CD) compared to its performance on true negatives.

Fusion and Ensemble models achieved both true negatives and true positives above 0.85, showing an increase of at least 0.08 in true positive compared to DNN. Among these models, Ensemble 1, fusing DNN2 and CNN1 with ensemble ML classifier, can be considered as the optimal model with an average F1-score 0.88, true negatives 0.88, and true positive 0.88.

### Interpretability analysis

**Fig 3:**
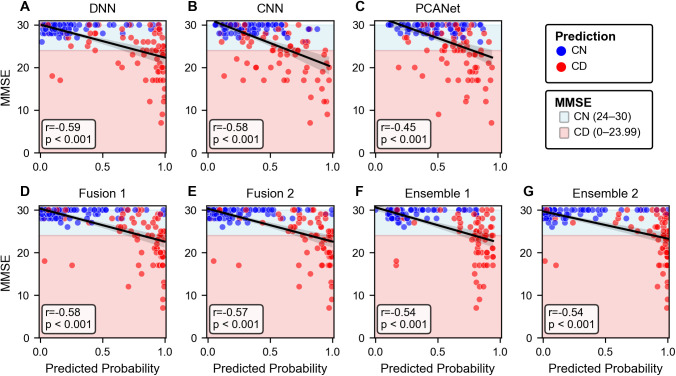
Correlation between MMSE and predicted probabilities from each model.

Moreover, the correlation analysis between MMSE and predicted probabilities from each model was conducted on test set (Fig 3). Total 147 subjects of out 252 subjects in all test set during 5-fold cross validation were included. From the analysis, we observed moderate negative relations (−0.8 < r < −0.5) except the probabilities from PCANet, having weak correlation. However, since all correlations have p < 0.001, there is a significant linear relationship between MMSE scores and predicted probabilities.

In the classification comparison between MMSE and our optimal model (Ensemble 1), Ensemble model shows higher performance except precision across the metrics (Table 5). MMSE has strong ability in correctly identifying CN (higher precision), while our model demonstrates superior performance in correctly detecting CD (higher recall). Similar results were obtained when performance was summarized as the average across folds (S9 Table).

**Table 5:** Comparison of classification by optimal model and MMSE.

Model	Accuracy	Precision	Recall	F1-Score	AUC
Ensemble 1	0.88	0.86	0.89	0.88	0.91
MMSE	0.72	1.00	0.42	0.59	

## Discussion

Although many recent studies on Alzheimer’s disease diagnosis have focused on MRI due to its higher spatial resolution, PET provides complementary molecular-level information that reflects underlying disease processes such as glucose metabolism. This makes PET particularly valuable for identifying cognitive decline, even when structural changes are less pronounced. In our study, we developed a deep learning framework that leverages complementary representation derived from the same FDG-PET modality by integrating voxel-level image features with region-level PET measurements. While voxel-level representations capture spatially distributed metabolic patterns, region-level measurements provide anatomically aggregated information that may improve robustness and interpretability. By validating this multi-scale single-modality representation framework across multiple feature dimensions and classification algorithms, we demonstrate that integrating complementary PET representations improve the classification performance, particularly sensitivity for detecting CD, a metric that is critical in clinical setting.

Fusion model with ensemble methods achieved the highest performance with accuracy of 0.87, recall of 0.88, and F1-score of 0.88. However, the tabular data based DNN still shows good performance with accuracy of 0.82 and it has the highest score in precision and AUC, which implies the regional SUVr has solid physiological information and also indicates the high contribution overall integration models. Nevertheless, the integration approaches still show the potential of improving in CD sensitivity (recall: 0.77 to 0.88) and more robust performance with lower standard deviation in 5-fold cross validation.

In the analysis with MMSE, there was a moderate relation between model prediction and MMSE scores. A higher correlation with MMSE did not necessarily indicate better classification performance, as Ensemble 1, which showed the best performance, did not have the highest correlation. This is because the CD has a wide range of MMSE, so even good CD detection could not have a high correlation with MMSE. Therefore, we examined the performance comparison with the diagnosis using MMSE. In the case of MMSE, the recall was relatively low while the precision was 1.00, suggesting that MMSE may fail to identify some CD cases. In clinical setting, the final diagnosis is made using multiple sources of information, not solely based on MMSE. The output of our model may therefore serve as a valuable diagnostic aid, particularly given its high sensitivity to CD compared to MMSE alone.

In our study, there are several limitations. First, many models are involved in our frameworks, so it is complicated to make the optimal choice for hyperparameters related to models and we compared the models in general setting. In other words, the performance could potentially be further improved through hyperparameter tuning, such as grid search or simulated annealing. Another limitation is the relatively small dataset, which reflects the practical constraints of PET-based research, where data availability is often limited due to acquisition cost and complexity [32,33]. Preprocessing steps such as MRI segmentation and regional SUVr extraction also introduce substantial workflow burden, which may limit scalability in broader clinical setting. Despite of these limitations, evaluating models under such data-constrained conditions remains practically important, as these constraints are commonly encountered in real-world clinical practice. To ensure a transparent and reliable performance evaluation on a small dataset, we employed 5-fold cross-validation and reported both the mean and standard deviation of model performance metrics across folds.

Although there are many advantages of PET, PET has lower resolution, and it is a challenging for PET-based model to achieving the higher accuracy comparing other modality (such as MRI) derived models. To tackle this challenging, several postprocessing strategies could be explored in future work to enhance resolution. Additionally, alternative feature concatenation methods, such as canonical correlation analysis (CCA) could be explored to further improve performance. Although CCA was tested in our experiments, it did not yield meaningful improvement, possibly due to suboptimal dimensional configurations. A more systematic tuning across dimensions may reveal its potential in future work.

## Conclusions

This study presented a deep learning framework that integrates complementary voxel-level and region-level representations derived from FDG PET data for cognitive decline classification. The proposed fusion framework achieved improved performance over single-representation models on the ADNI dataset, particularly in identifying cognitively impaired individuals. A moderate correlation with MMSE scores suggests alignment between model outputs and established cognitive assessments. These findings support the potential utility of multi-scale FDG-PET representations in machine learning–based cognitive decline detection, with implications for clinical diagnostic aid.

## Supporting information

S1 Table List of 41 regions considered for regional SUVr extraction. (DOCX)

S2 Table Summary of DNN architecture. (DOCX)

S3 Table Summary of CNN architecture. (DOCX)

S4 Table Summary of PCANet architecture. (DOCX)

S5 Table PCANet prediction results with different ML classifier. (DOCX)

S6 Table Comparison of fusion models with different feature levels with RF. (DOCX)

S7 Table Comparison of fusion models with different ML classifiers using DNN2 and CNN1 features. (DOCX)

S8 Table Comparison of models with different metrics. (DOCX)

S9 Table Comparison of classification by models and MMSE. (DOCX)
