# Supplementary material for: Feature integration of [18F]FDG PET brain imaging using deep learning for sensitive cognitive decline detection
Source: PLoS One. 2026 Jul 21;21(7):e0341995. doi: 10.1371/journal.pone.0341995 (PMC13387574; doi:10.1371/journal.pone.0341995)
Supplement: S1 Table — (DOCX) [file pone.0341995.s001.docx]

**S1 Table. List of 41 regions considered for regional SUVr extraction.**

| **Regional SUVr Features** | | | |
| --- | --- | --- | --- |
| No. | Region Label | No. | Region Label |
| 2 | Left-Cerebral-White-Matter | 41 | Right-Cerebral-White-Matter |
| 3 | Left-Cerebral-Cortex | 42 | Right-Cerebral-Cortex |
| 4 | Left-Lateral-Ventricle | 43 | Right-Lateral-Ventricle |
| 5 | Left-Inf-Lat-Vent | 44 | Right-Inf-Lat-Vent |
| 7 | Left-Cerebellum-White-Matter | 46 | Right-Cerebellum-White-Matter |
| 8 | Left-Cerebellum-Cortex | 47 | Right-Cerebellum-Cortex |
| 10 | Left-Thalamus-Proper | 49 | Right-Thalamus-Proper |
| 11 | Left-Caudate | 50 | Right-Caudate |
| 12 | Left-Putamen | 51 | Right-Putamen |
| 13 | Left-Pallidum | 52 | Right-Pallidum |
| 14 | 3rd-Ventricle | 53 | Right-Hippocampus |
| 15 | 4th-Ventricle | 54 | Right-Amygdala |
| 16 | Brain-Stem | 58 | Right-Accumbens-area |
| 17 | Left-Hippocampus | 60 | Right-VentralDC |
| 18 | Left-Amygdala | 63 | Right-choroid-plexus |
| 24 | CSF | 77 | WM-hypointensities |
| 26 | Left-Accumbens-area | 85 | Optic-Chiasm |
| 28 | Left-VentralDC | 251 | CC_Posterior |
| 31 | Left-choroid-plexus | 252 | CC_Mid_Posterior |
|  |  | 253 | CC_Central |
|  |  | 254 | CC_Mid_Anterior |
|  |  | 255 | CC_Anterior |

The label numbers correspond to region indices defined by the FreeSurfer segmentation protocol.
