# Supplementary material for: Feature integration of [18F]FDG PET brain imaging using deep learning for sensitive cognitive decline detection
Source: PLoS One. 2026 Jul 21;21(7):e0341995. doi: 10.1371/journal.pone.0341995 (PMC13387574; doi:10.1371/journal.pone.0341995)
Supplement: S2 Table — (DOCX) [file pone.0341995.s002.docx]

**S2 Table. Summary of DNN Architecture.**

| **Layer** | **Activation Function** | **Output Shape** | **# of Param** |
| --- | --- | --- | --- |
| Input Layer |  | 41 | 0 |
| Dense Layer | ReLu | 64 | 2,688 |
| Batch Normalization Layer |  | 64 | 256 |
| Dropout Value (0.3) |  | 64 | 0 |
| Dense Layer | ReLu | 32 | 2,080 |
| Batch Normalization Layer |  | 32 | 128 |
| Dropout Value (0.3) |  | 32 | 0 |
| Dense Layer | ReLu | 16 | 528 |
| Batch Normalization Layer |  | 16 | 64 |
| Dropout Value (0.3) |  | 16 | 0 |
| Dense Layer | Sigmoid | 1 | 17 |
