# Supplementary material for: Feature integration of [18F]FDG PET brain imaging using deep learning for sensitive cognitive decline detection
Source: PLoS One. 2026 Jul 21;21(7):e0341995. doi: 10.1371/journal.pone.0341995 (PMC13387574; doi:10.1371/journal.pone.0341995)
Supplement: S4 Table — (DOCX) [file pone.0341995.s004.docx]

**S4 Table. Summary of PCANet Architecture.**

| Layer | Filter Shape | Step Shape | # of Filters | Output Shape |
| --- | --- | --- | --- | --- |
| PCA Convolution Layer | 16×16 | 4×4 | 8 | (29, 29, 8) |
| PCA Convolution Layer | 16×16 | 4×4 | 4 | (4, 4, 32) |
| Pooling Layer | 4×4 | 2×2 |  | 128 |
