# Supplementary material for: Feature integration of [18F]FDG PET brain imaging using deep learning for sensitive cognitive decline detection
Source: PLoS One. 2026 Jul 21;21(7):e0341995. doi: 10.1371/journal.pone.0341995 (PMC13387574; doi:10.1371/journal.pone.0341995)
Supplement: S5 Table — (DOCX) [file pone.0341995.s005.docx]

**S5 Table. PCANet prediction results with different ML classifier.**

| **Classifier** | **Accuracy** | **Precision** | **Recall** | **F1-Score** | **AUC** |
| --- | --- | --- | --- | --- | --- |
| CB | 0.66 ± 0.04 | 0.66 ± 0.03 | 0.77 ± 0.06 | 0.71 ± 0.04 | 0.65 ± 0.04 |
| DT | 0.57 ± 0.07 | 0.60 ± 0.07 | 0.57 ± 0.08 | 0.59 ± 0.07 | 0.57 ± 0.07 |
| ET | 0.65 ± 0.05 | 0.64 ± 0.03 | **0.78 ± 0.08** | 0.71 ± 0.04 | 0.65 ± 0.05 |
| GB | 0.65 ± 0.04 | 0.66 ± 0.03 | 0.74 ± 0.07 | 0.69 ± 0.04 | 0.65 ± 0.04 |
| KNN | 0.58 ± 0.03 | 0.62 ± 0.02 | 0.57 ± 0.10 | 0.59 ± 0.06 | 0.58 ± 0.03 |
| LGBM | 0.65 ± 0.05 | 0.65 ± 0.03 | 0.72 ± 0.08 | 0.68 ± 0.05 | 0.64 ± 0.04 |
| LR | 0.64 ± 0.08 | 0.67 ± 0.07 | 0.66 ± 0.11 | 0.66 ± 0.08 | 0.64 ± 0.08 |
| MLP | 0.67 ± 0.07 | 0.68 ± 0.08 | 0.72 ± 0.06 | 0.70 ± 0.06 | 0.66 ± 0.08 |
| NB | 0.56 ± 0.02 | 0.62 ± 0.06 | 0.52 ± 0.22 | 0.54 ± 0.10 | 0.56 ± 0.02 |
| RF | 0.66 ± 0.05 | 0.65 ± 0.02 | **0.78 ± 0.08** | 0.71 ± 0.05 | 0.65 ± 0.05 |
| SVM | 0.65 ± 0.07 | 0.68 ± 0.05 | 0.67 ± 0.14 | 0.67 ± 0.08 | 0.65 ± 0.07 |
| XGB | **0.69 ± 0.06** | **0.69 ± 0.05** | 0.75 ± 0.08 | **0.72 ± 0.05** | **0.69 ± 0.06** |
