# Supplementary material for: Feature integration of [18F]FDG PET brain imaging using deep learning for sensitive cognitive decline detection
Source: PLoS One. 2026 Jul 21;21(7):e0341995. doi: 10.1371/journal.pone.0341995 (PMC13387574; doi:10.1371/journal.pone.0341995)
Supplement: S6 Table — (DOCX) [file pone.0341995.s006.docx]

**S6 Table. Comparison of fusion models with different feature levels with RF.**

| Regional SUVr | PET imaging | Accuracy | Precision | Recall | F1-Score | AUC |
| --- | --- | --- | --- | --- | --- | --- |
| DNN0 | PCANet | 0.82 ± 0.08 | 0.83 ± 0.10 | 0.84 ± 0.09 | 0.84 ± 0.07 | 0.82 ± 0.08 |
| DNN0 | CNN1 | 0.80 ± 0.05 | 0.82 ± 0.08 | 0.81 ± 0.05 | 0.81 ± 0.04 | 0.80 ± 0.05 |
| DNN0 | CNN2 | 0.81 ± 0.04 | 0.83 ± 0.08 | 0.81 ± 0.05 | 0.82 ± 0.04 | 0.81 ± 0.05 |
| DNN0 | CNN3 | 0.78 ± 0.06 | 0.82 ± 0.05 | 0.75 ± 0.10 | 0.78 ± 0.06 | 0.78 ± 0.05 |
| DNN1 | PCANet | 0.83 ± 0.07 | 0.85 ± 0.09 | 0.83 ± 0.06 | 0.84 ± 0.06 | 0.82 ± 0.07 |
| DNN1 | CNN1 | 0.84 ± 0.06 | 0.86 ± 0.08 | 0.85 ± 0.04 | 0.85 ± 0.05 | 0.84 ± 0.06 |
| DNN1 | CNN2 | 0.85 ± 0.05 | 0.87 ± 0.09 | 0.86 ± 0.03 | 0.86 ± 0.05 | 0.85 ± 0.06 |
| DNN1 | CNN3 | 0.84 ± 0.06 | 0.86 ± 0.08 | 0.84 ± 0.06 | 0.85 ± 0.06 | 0.84 ± 0.06 |
| DNN2 | PCANet | 0.83 ± 0.06 | 0.85 ± 0.09 | 0.85 ± 0.04 | 0.85 ± 0.05 | 0.83 ± 0.06 |
| DNN2 | CNN1 | **0.86 ± 0.05** | 0.88 ± 0.07 | 0.86 ± 0.03 | **0.87 ± 0.04** | **0.86 ± 0.05** |
| DNN2 | CNN2 | **0.86 ± 0.06** | **0.89 ± 0.09** | 0.86 ± 0.03 | **0.87 ± 0.05** | **0.86 ± 0.06** |
| DNN2 | CNN3 | 0.85 ± 0.06 | 0.88 ± 0.08 | 0.84 ± 0.07 | 0.86 ± 0.06 | 0.85 ± 0.06 |
| DNN3 | PCANet | 0.82 ± 0.06 | 0.81 ± 0.09 | **0.87 ± 0.05** | 0.84 ± 0.05 | 0.81 ± 0.07 |
| DNN3 | CNN1 | 0.83 ± 0.06 | 0.86 ± 0.09 | 0.83 ± 0.06 | 0.84 ± 0.05 | 0.83 ± 0.06 |
| DNN3 | CNN2 | 0.84 ± 0.06 | 0.86 ± 0.09 | 0.84 ± 0.06 | 0.85 ± 0.06 | 0.84 ± 0.06 |
| DNN3 | CNN3 | 0.81 ± 0.05 | 0.83 ± 0.08 | 0.81 ± 0.10 | 0.82 ± 0.05 | 0.80 ± 0.05 |

Bold text: the highest average value for each metric
