# Supplementary material for: Feature integration of [18F]FDG PET brain imaging using deep learning for sensitive cognitive decline detection
Source: PLoS One. 2026 Jul 21;21(7):e0341995. doi: 10.1371/journal.pone.0341995 (PMC13387574; doi:10.1371/journal.pone.0341995)
Supplement: S7 Table — (DOCX) [file pone.0341995.s007.docx]

**S7 Table. Comparison of fusion models with different ML classifiers using DNN2 and CNN1 features.**

| **Classifier** | **Accuracy** | **Precision** | **Recall** | **F1-Score** | **AUC** |
| --- | --- | --- | --- | --- | --- |
| CB | 0.85 ± 0.06 | **0.88 ± 0.10** | 0.85 ± 0.07 | 0.86 ± 0.05 | 0.85 ± 0.06 |
| DT | 0.75 ± 0.04 | 0.76 ± 0.05 | 0.78 ± 0.04 | 0.77 ± 0.03 | 0.75 ± 0.04 |
| ET | **0.86 ± 0.04** | **0.88 ± 0.07** | 0.85 ± 0.03 | 0.86 ± 0.03 | **0.86 ± 0.04** |
| GB | 0.81 ± 0.07 | 0.82 ± 0.09 | 0.84 ± 0.08 | 0.83 ± 0.07 | 0.81 ± 0.08 |
| KNN | 0.80 ± 0.03 | 0.87 ± 0.06 | 0.75 ± 0.10 | 0.80 ± 0.05 | 0.81 ± 0.03 |
| LGBM | 0.82 ± 0.06 | 0.83 ± 0.08 | 0.84 ± 0.06 | 0.84 ± 0.05 | 0.82 ± 0.06 |
| LR | 0.82 ± 0.04 | 0.84 ± 0.05 | 0.81 ± 0.07 | 0.83 ± 0.04 | 0.82 ± 0.04 |
| MLP | 0.85 ± 0.03 | 0.86 ± 0.04 | **0.86 ± 0.04** | 0.86 ± 0.03 | 0.85 ± 0.03 |
| NB | 0.83 ± 0.04 | **0.88 ± 0.08** | 0.79 ± 0.04 | 0.83 ± 0.03 | 0.83 ± 0.04 |
| RF  (Fusion 1) | **0.86 ± 0.05** | **0.88 ± 0.07** | **0.86 ± 0.03** | **0.87 ± 0.04** | **0.86 ± 0.05** |
| SVM | 0.83 ± 0.07 | 0.85 ± 0.09 | 0.83 ± 0.07 | 0.84 ± 0.07 | 0.82 ± 0.07 |
| XGB | 0.82 ± 0.07 | 0.84 ± 0.10 | 0.84 ± 0.08 | 0.84 ± 0.06 | 0.82 ± 0.07 |

Bold text: the highest average value for each metric
