# Supplementary material for: Feature integration of [18F]FDG PET brain imaging using deep learning for sensitive cognitive decline detection
Source: PLoS One. 2026 Jul 21;21(7):e0341995. doi: 10.1371/journal.pone.0341995 (PMC13387574; doi:10.1371/journal.pone.0341995)
Supplement: S8 Table — (DOCX) [file pone.0341995.s008.docx]

**S8 Table. Comparison of models with different metrics.**

| Regional SUVr | PET imaging | Ensemble | Accuracy | Precision | Recall | F1-Score | AUC |
| --- | --- | --- | --- | --- | --- | --- | --- |
| DNN2 | CNN1 | Soft Voting | 0.86 ± 0.06 | 0.87 ± 0.08 | **0.88 ± 0.04** | 0.87 ± 0.05 | 0.87 ± 0.04 |
|  |  | **Stacking**  **(Ensemble 1)** | **0.87 ± 0.05** | **0.89 ± 0.06** | **0.88 ± 0.04** | **0.88 ± 0.05** | **0.88 ± 0.04** |
|  |  | Hard Voting | 0.86 ± 0.05 | 0.86 ± 0.06 | **0.88 ± 0.04** | 0.87 ± 0.04 | 0.86 ± 0.05 |
|  | CNN2 | **Soft Voting**  **(Ensemble 2)** | **0.87 ± 0.05** | 0.88 ± 0.07 | **0.88 ± 0.04** | **0.88 ± 0.05** | 0.87 ± 0.05 |
|  |  | Stacking | 0.86 ± 0.06 | 0.87 ± 0.07 | 0.87 ± 0.04 | 0.87 ± 0.05 | 0.87 ± 0.04 |
|  |  | Hard Voting | 0.86 ± 0.06 | 0.88 ± 0.07 | 0.87 ± 0.06 | 0.87 ± 0.05 | 0.86 ± 0.06 |

Bold text: the highest average value for each metric
