# Supplementary material for: Feature integration of [18F]FDG PET brain imaging using deep learning for sensitive cognitive decline detection
Source: PLoS One. 2026 Jul 21;21(7):e0341995. doi: 10.1371/journal.pone.0341995 (PMC13387574; doi:10.1371/journal.pone.0341995)
Supplement: S9 Table — (DOCX) [file pone.0341995.s009.docx]

**S9 Table. Comparison of classification by models and MMSE.**

Model performance evaluated on all available test predictions (“All”, n=147), and performance averaged across 5-fold cross-validation (“Fold-average”, n=29, 29, 28, 27, and 34). Only patients with MMSE were used for comparisons.

| **All** | | | | | |
| --- | --- | --- | --- | --- | --- |
| **Model** | **Accuracy** | **Precision** | **Recall** | **F1-Score** | **AUC** |
| CNN | 0.71 | 0.75 | 0.62 | 0.68 | 0.75 |
| DNN | 0.81 | 0.83 | 0.76 | 0.79 | 0.88 |
| PCANet | 0.71 | 0.67 | 0.82 | 0.73 | 0.76 |
| Fusion 1 | 0.85 | 0.83 | 0.87 | 0.85 | 0.88 |
| Fusion 2 | 0.86 | 0.84 | 0.87 | 0.86 | 0.88 |
| Ensemble 1 | 0.88 | 0.86 | 0.89 | 0.88 | 0.91 |
| Ensemble 2 | 0.87 | 0.85 | 0.89 | 0.87 | 0.88 |
| MMSE | 0.72 | 1 | 0.42 | 0.59 |  |
| **Fold-average** | | | | | |
| **Model** | **Accuracy** | **Precision** | **Recall** | **F1-Score** | **AUC** |
| CNN | 0.71 ± 0.08 | 0.75 ± 0.11 | 0.62 ± 0.06 | 0.68 ± 0.07 | 0.75 ± 0.05 |
| DNN | 0.81 ± 0.10 | 0.85 ± 0.13 | 0.77 ± 0.21 | 0.79 ± 0.14 | 0.90 ± 0.06 |
| PCANet | 0.72 ± 0.11 | 0.67 ± 0.11 | 0.83 ± 0.15 | 0.74 ± 0.11 | 0.78 ± 0.12 |
| Fusion 1 | 0.85 ± 0.07 | 0.84 ± 0.13 | 0.88 ± 0.08 | 0.85 ± 0.07 | 0.89 ± 0.05 |
| Fusion 2 | 0.86 ± 0.08 | 0.86 ± 0.15 | 0.88 ± 0.08 | 0.86 ± 0.08 | 0.89 ± 0.04 |
| Ensemble 1 | 0.88 ± 0.05 | 0.87 ± 0.10 | 0.89 ± 0.07 | 0.88 ± 0.05 | 0.89 ± 0.03 |
| Ensemble 2 | 0.87 ± 0.05 | 0.86 ± 0.11 | 0.89 ± 0.07 | 0.87 ± 0.06 | 0.89 ± 0.04 |
| MMSE | 0.72 ± 0.08 | 1.00 ± 0.00 | 0.42 ± 0.15 | 0.58 ± 0.15 |  |
