# Supplementary material for: Feature integration of [18F]FDG PET brain imaging using deep learning for sensitive cognitive decline detection
Source: PLoS One. 2026 Jul 21;21(7):e0341995. doi: 10.1371/journal.pone.0341995 (PMC13387574; doi:10.1371/journal.pone.0341995)
Supplement: Attachment — Submitted filename: Response to Reviewers.docx [file pone.0341995.s011.docx]

June 17, 2026

Dear Editor,

We would like to thank the reviewers for their insightful comments on our manuscript titled “*Feature Integration of [^18^F]FDG PET Brain Imaging Using Deep Learning for Sensitive Cognitive Decline Detection*” (PONE-D-26-02360). We have carefully considered each point raised and made the necessary revisions to the manuscript. Below, we provide a detailed response to each comment.

**Editor Comments**

**Comment 1: Manuscript formatting and PLOS ONE style requirements**

**Response**: We have revised the manuscript formatting in accordance with PLOS ONE style requirements, including Supporting Information file naming, title formatting, citations, and the symbol notation used for the consortium.

**Comment 2: Code sharing policy**

**Response**: We thank the reviewer for this suggestion. In accordance with PLOS ONE’s code sharing policy, we have deposited the author-generated code in a publicly accessible GitHub repository to facilitate reproducibility and reuse.

Repository: <https://github.com/leeyoujin0221/feature_integration_AD>

We have revised the Data Availability Statement accordingly and included the repository link in the manuscript.

**Comment 3 and 5: ADNI consortium author information**

**Response**: Thank you for this comment. The Alzheimer’s Disease Neuroimaging Initiative (ADNI) includes a very large number of contributors across multiple institutions. Following common practice in ADNI-based publications, we have acknowledged the consortium as a group author and provided a reference to the official ADNI Acknowledgement List rather than listing the individual authors in the manuscript. This approach has also been adopted in recent ADNI-based publications, including Gonzales et al. (2026), “White matter microstructure disruption associated with PET and cognitive impairment in Alzheimer’s disease” (PLOS ONE, 21(4): e0346661; <https://doi.org/10.1371/journal.pone.0346661>).

Accordingly, we have revised the Acknowledgments section to include the official ADNI Acknowledgement List (<https://adni.loni.usc.edu/wp-content/uploads/how_to_apply/ADNI_Acknowledgement_List.pdf>).

**Comment 4: Funding statement correction**

**Response**: We removed funding-related text from the manuscript in accordance with PLOS ONE policy and provided the corrected Funding Statement in the cover letter for updating the online submission record.

**Comment 6: Figure 1 copyright compliance**

**Response**: We contacted ADNI regarding copyright permission for the representative ADNI-derived image included in Figure 1 and obtained written permission for publication under the CC BY 4.0 license. The signed permission form has been provided as supporting documentation.

**Comment 8: Reviewer-suggested references**

**Response:** Thank you for this comment. We reviewed the reviewer comments and confirmed that no specific recommendations for additional citations were provided.

**Reviewer 1**

Thank you for your comments. We have revised the manuscript to improve its clarity and readability. We also updated the reported improvement percentage in the Abstract by recalculating it using the original (unrounded) values rather than the rounded values shown in the table; this update does not affect the reported performance values or the conclusions of the study. Each comment is addressed in detail below.

**Major 1.** Page 4, lines 83-85. The statement that FDG-PET plays a key role in the early detection of AD seems factually imprecise and potentially misleading. FDG-PET is better considered a marker of downstream neurodegenerative/metabolic change, whereas amyloid and tau biomarkers are more directly tied to early AD pathology.

More broadly, the manuscript appears to repeatedly attribute to FDG-PET some of the molecular specificity advantages that are more characteristic of other PET tracers or biomarker frameworks. The authors should carefully re-examine the wording throughout the manuscript and revise statements that overgeneralize PET’s molecular specificity in a way that does not accurately apply to FDG-PET.

**Response**: We thank the reviewer for this important comment. We agree that FDG-PET primarily reflects downstream neurodegenerative and metabolic changes rather than early AD-specific molecular pathology. Accordingly, we carefully reviewed and revised the relevant statements throughout the manuscript to avoid overstating the molecular specificity or early detection role of FDG-PET.

Specifically, the statement has been revised as follows (Page 4, lines 80-82): “Among current diagnostic tools, positron emission tomography (PET) plays an important role in the evaluation of AD, with different tracers providing complementary diagnostic information [17-19].” The corresponding references have also been updated accordingly.

**Major 2.** Page 5, lines 108-112. The logic in this paragraph is unclear. The authors first highlight inter-institutional variability in scanner resolution and imaging protocols as a key limitation to generalizability, but then shift to the need for models that perform well on small datasets. These are not equivalent issues. Good performance in small datasets does not by itself address cross-scanner, cross-site, or protocol-related heterogeneity. The authors should clarify this rationale and avoid implying that limited-data modeling alone can overcome institutional variability in PET acquisition.

**Response**: We thank the reviewer for this helpful comment. We agree that our original wording incorrectly linked inter-institutional acquisition variability with limited-data model performance, which are distinct challenges. Our intended point was that PET imaging is costly and requires radioactive tracers, which restrict large-scale data acquisition and result in relatively limited datasets for model development. We have revised the paragraph accordingly to clarify this rationale.

Specifically, the statement has been revised as follows (Page 5, lines 106-110): “However, PET imaging requires radioactive tracers and dedicated imaging infrastructure, making data acquisition expensive and comparatively limited relative to other imaging modalities [33, 34]. Consequently, developing models that can achieve robust performance with limited training data may enhance the clinical utility of PET-based diagnostic tools.” The corresponding references have also been updated accordingly.

**Major 3.** Page 9, lines 180-185. The term “quantification” appears imprecise here. As described, the analysis involves extraction/calculation of regional SUVr values rather than quantitative PET modeling. This terminology may overstate the nature of the PET analysis and should be revised accordingly. Because similar wording appears elsewhere in the manuscript, the authors should review and correct this terminology throughout the paper for greater methodological precision.

**Response**: We thank the reviewer for this important comment. We agree that the term “quantification” may overstate the nature of our PET analysis, as our approach involved extraction of regional PET measurements (SUVr values) rather than formal quantitative PET modeling. Accordingly, we revised the terminology throughout the manuscript for greater methodological precision, replacing “quantification” with context-appropriate terms such as “region-level PET measurements” and “regional SUVr values,” and clarifying that SUVr represents a semi-quantitative PET measure.

Specifically, the statement has been revised as follows (Page 9, lines 178-183): “Another limitation is the relatively small dataset, which reflects the practical constraints of PET-based research, where data availability is often limited due to acquisition cost and complexity [33, 34]. Preprocessing steps such as MRI segmentation and regional SUVr extraction also introduce substantial workflow burden, which may limit scalability in broader clinical setting. Despite of these limitations, evaluating models under such data-constrained conditions remains practically important, as these constraints are commonly encountered in real-world clinical practice.”

**Major 4.** Page 18, lines 367-370. The wording here is somewhat vague and may overstate the methodological novelty. As described in the Methods, both the image-derived and region-based features are derived from the same FDG-PET data, with the latter representing ROI-level SUVr values. Thus, the framework appears to combine two representations of the same modality rather than fundamentally different data sources. The authors should clarify this point and describe more explicitly how these complementary representations provide additional information.

**Response**: We thank the reviewer for this insightful comment. We agree that our framework integrates complementary representations derived from the same FDG-PET modality rather than fundamentally different data sources. Our intention was to emphasize the complementary nature of multi-scale representations rather than multimodal integration. We have revised the text to clarify that voxel-level image features capture distributed spatial metabolic patterns, whereas region-level PET measurements provide anatomically summarized information that may offer complementary discriminatory value.

Specifically, the statement has been revised as follows (Page 18, lines 364-369): “In our study, we developed a deep learning framework that leverages complementary representation derived from the same FDG-PET modality by integrating voxel-level image features with region-level PET measurements. While voxel-level representations capture spatially distributed metabolic patterns, region-level measurements provide anatomically aggregated information that may improve robustness and interpretability.”

**Major 5.** Page 20, Conclusion. The Conclusion appears somewhat overstated relative to the actual scope of the study. The reported results mainly show improved internal classification performance within ADNI when combining two FDG-PET-derived representations, rather than establishing early detection capability, clinical relevance, or superior disease differentiability in a broader diagnostic sense. In particular, the statements regarding “early detection of Alzheimer’s disease,” MMSE correlation “confirming” clinical relevance, and better disease differentiability compared with MMSE should be toned down. The conclusion would be more appropriate if it emphasized improved comparative model performance within this dataset and framed the clinical implications more cautiously.

**Response**: We thank the reviewer for this thoughtful comment. We agree that several statements in the original conclusion overstated the scope of our findings. Our results demonstrate improved comparative classification performance within the ADNI dataset rather than establishing broader clinical diagnostic utility or early detection capability. Accordingly, we revised the conclusion to adopt a more cautious interpretation, clarifying that MMSE correlations indicate consistency with cognitive measures rather than confirming clinical relevance.

Specifically, conclusion has been revised as follows (Page 20): “This study presented a deep learning framework that integrates complementary voxel-level and region-level representations derived from FDG PET data for cognitive decline classification. The proposed fusion framework achieved improved performance over single-representation models on the ADNI dataset, particularly in identifying cognitively impaired individuals. A moderate correlation with MMSE scores suggests alignment between model outputs and established cognitive assessments. These findings support the potential utility of multi-scale FDG-PET representations in machine learning–based cognitive decline detection, with implications for clinical diagnostic aid.”

**Minor 1.** Page 19, lines 397-399. This statement is unclear. The need for segmentation and region-based SUVR extraction does not by itself explain why only a small dataset was available, nor why such a dataset would better reflect real-world clinical settings. Please clarify the reason for the reduced sample size.

**Response**: We thank the reviewer for this helpful comment. We agree that our original wording did not clearly explain the rationale for the limited dataset size. Our intention was not to suggest that segmentation or SUVr extraction directly determined sample availability, but rather that PET-based studies often face practical data constraints due to the high cost of PET imaging, the requirement for radioactive tracers, and the time-intensive preprocessing workflow (e.g., MRI segmentation and regional SUVr extraction). We have revised the text accordingly to clarify this point.

Specifically, the statement has been revised as follows (Page 19, lines 397-404): “Another limitation is the relatively small dataset, which reflects the practical constraints of PET-based research, where data availability is often limited due to acquisition cost and complexity [33, 34]. Preprocessing steps such as MRI segmentation and regional SUVr extraction also introduce substantial workflow burden, which may limit scalability in broader clinical setting. Despite of these limitations, evaluating models under such data-constrained conditions remains practically important, as these constraints are commonly encountered in real-world clinical practice.”
