# Supplementary material for: Feature integration of [18F]FDG PET brain imaging using deep learning for sensitive cognitive decline detection
Source: PLoS One. 2026 Jul 21;21(7):e0341995. doi: 10.1371/journal.pone.0341995 (PMC13387574; doi:10.1371/journal.pone.0341995)
Supplement: Peer review history

# Peer review: Decision Letter 0

**DOI:** 10.1371/journal.pone.0341995.r001

**Authors:** Emanuele Bartolini

6 May 2026

PONE-D-26-02360

Feature Integration of [18F]FDG PET Brain Imaging Using Deep Learning for Sensitive Cognitive Decline Detection

PLOS One

Dear Dr. Kang,

Thank you for submitting your manuscript to PLOS ONE. After careful consideration, we feel that it has merit but does not fully meet PLOS ONE’s publication criteria as it currently stands. Therefore, we invite you to submit a revised version of the manuscript that addresses the points raised during the review process.

Please submit your revised manuscript by Jun 20 2026 11:59PM. If you will need more time than this to complete your revisions, please reply to this message or contact the journal office at plosone@plos.org. When you're ready to submit your revision, log on to https://www.editorialmanager.com/pone/ and select the 'Submissions Needing Revision' folder to locate your manuscript file.

Please include the following items when submitting your revised manuscript:

- A letter that responds to each point raised by the academic editor and reviewer(s). You should upload this letter as a separate file labeled 'Response to Reviewers'.
- A marked-up copy of your manuscript that highlights changes made to the original version. You should upload this as a separate file labeled 'Revised Manuscript with Track Changes'.
- An unmarked version of your revised paper without tracked changes. You should upload this as a separate file labeled 'Manuscript'.

If you would like to make changes to your financial disclosure, please include your updated statement in your cover letter. Guidelines for resubmitting your figure files are available below the reviewer comments at the end of this letter.

If applicable, we recommend that you deposit your laboratory protocols in protocols.io to enhance the reproducibility of your results. Protocols.io assigns your protocol its own identifier (DOI) so that it can be cited independently in the future. For instructions see: https://journals.plos.org/plosone/s/submission-guidelines#loc-laboratory-protocols. Additionally, PLOS ONE offers an option for publishing peer-reviewed Lab Protocol articles, which describe protocols hosted on protocols.io. Read more information on sharing protocols at https://plos.org/protocols?utm_medium=editorial-email&utm_source=authorletters&utm_campaign=protocols.

As the corresponding author, your ORCID iD is verified in the submission system and will appear in the published article. PLOS supports the use of ORCID, and we encourage all coauthors to register for an ORCID iD and use it as well. Please encourage your coauthors to verify their ORCID iD within the submission system before final acceptance, as unverified ORCID iDs will not appear in the published article. *Only*  the individual author can complete the verification step; PLOS staff *cannot*  verify ORCID iDs on behalf of authors.

We look forward to receiving your revised manuscript.

Kind regards,

Emanuele Bartolini, MD

Academic Editor

PLOS One

Journal Requirements:

When submitting your revision, we need you to address these additional requirements.

1. Please ensure that your manuscript meets PLOS ONE's style requirements, including those for file naming. The PLOS ONE style templates can be found at

https://journals.plos.org/plosone/s/file?id=wjVg/PLOSOne_formatting_sample_main_body.pdf and https://journals.plos.org/plosone/s/file?id=ba62/PLOSOne_formatting_sample_title_authors_affiliations.pdf

2. Please note that PLOS One has specific guidelines on code sharing for submissions in which author-generated code underpins the findings in the manuscript. In these cases, we expect all author-generated code to be made available without restrictions upon publication of the work. Please review our guidelines at https://journals.plos.org/plosone/s/materials-and-software-sharing#loc-sharing-code and ensure that your code is shared in a way that follows best practice and facilitates reproducibility and reuse.

3. One of the noted authors is a group or consortium [Alzheimer’s Disease Neuroimaging Initiative]. In addition to naming the author group, please list the individual authors and affiliations within this group in the acknowledgments section of your manuscript. Please also indicate clearly a lead author for this group along with a contact email address.

4. Thank you for stating the following in the Acknowledgments Section of your manuscript:

Data collection and sharing for this project was funded by the Alzheimer's Disease Neuroimaging Initiative (ADNI) (National Institutes of Health Grant U01 AG024904) and DOD ADNI (Department of Defense award number W81XWH-12-2-0012). ADNI is funded by the National Institute on Aging, the National Institute of Biomedical Imaging and Bioengineering, and through generous contributions from the following: AbbVie, Alzheimer’s Association; Alzheimer’s Drug Discovery Foundation; Araclon Biotech; BioClinica, Inc.; Biogen; Bristol-Myers Squibb Company; CereSpir, Inc.; Cogstate; Eisai Inc.; Elan Pharmaceuticals, Inc.; Eli Lilly and Company; EuroImmun; F. Hoffmann-La Roche Ltd and its affiliated company Genentech, Inc.; Fujirebio; GE Healthcare; IXICO Ltd.; Janssen Alzheimer Immunotherapy Research & Development, LLC.; Johnson & Johnson Pharmaceutical Research & Development LLC.; Lumosity; Lundbeck; Merck & Co., Inc.; Meso Scale Diagnostics, LLC.; NeuroRx Research; Neurotrack Technologies; Novartis Pharmaceuticals Corporation; Pfizer Inc.; Piramal Imaging; Servier; Takeda Pharmaceutical Company; and Transition Therapeutics. The Canadian Institutes of Health Research is providing funds to support ADNI clinical sites in Canada. Private sector contributions are facilitated by the Foundation for the National Institutes of Health (www.fnih.org). The grantee organization is the Northern California Institute for Research and Education, and the study is coordinated by the Alzheimer’s Therapeutic Research Institute at the University of Southern California. ADNI data are disseminated by the Laboratory for Neuro Imaging at the University of Southern 460 California.

We note that you have provided funding information that is not currently declared in your Funding Statement. However, funding information should not appear in the Acknowledgments section or other areas of your manuscript. We will only publish funding information present in the Funding Statement section of the online submission form.

Please remove any funding-related text from the manuscript and let us know how you would like to update your Funding Statement. Currently, your Funding Statement reads as follows:

This material is based upon work supported by the Air Force Office of Scientific Research under Award No. FA9550-22-1-0272 (YK). Also, this work was supported by the National Research Foundation of Korea(NRF) grant funded by the Korea government(MSIT) (RS-2024-00406152) (YL). Also, this work was supported by Global - Learning & Academic research institution for Master’s, PhD students, and Postdocs (LAMP) Program of the National Research Foundation of Korea (NRF) grant funded by the Ministry of Education (No. RS-2023-00301938) (SK).

Please include your amended statements within your cover letter; we will change the online submission form on your behalf.

5. One of the noted authors is a group or consortium [Alzheimer’s Disease Neuroimaging Initiative]. In addition to naming the author group, please list the individual authors and affiliations within this group in the acknowledgments section of your manuscript. Please also indicate clearly a lead author for this group along with a contact email address.

6. We note that Figure 1 in your submission contain copyrighted images. All PLOS content is published under the Creative Commons Attribution License (CC BY 4.0), which means that the manuscript, images, and Supporting Information files will be freely available online, and any third party is permitted to access, download, copy, distribute, and use these materials in any way, even commercially, with proper attribution. For more information, see our copyright guidelines: http://journals.plos.org/plosone/s/licenses-and-copyright.

We require you to either (1) present written permission from the copyright holder to publish these figures specifically under the CC BY 4.0 license, or (2) remove the figures from your submission:

a. You may seek permission from the original copyright holder of Figure 1 to publish the content specifically under the CC BY 4.0 license.

We recommend that you contact the original copyright holder with the Content Permission Form (http://journals.plos.org/plosone/s/file?id=7c09/content-permission-form.pdf) and the following text:

“I request permission for the open-access journal PLOS ONE to publish XXX under the Creative Commons Attribution License (CCAL) CC BY 4.0 (http://creativecommons.org/licenses/by/4.0/). Please be aware that this license allows unrestricted use and distribution, even commercially, by third parties. Please reply and provide explicit written permission to publish XXX under a CC BY license and complete the attached form.”

Please upload the completed Content Permission Form or other proof of granted permissions as an "Other" file with your submission.

In the figure caption of the copyrighted figure, please include the following text: “Reprinted from [ref] under a CC BY license, with permission from [name of publisher], original copyright [original copyright year].”

b. If you are unable to obtain permission from the original copyright holder to publish these figures under the CC BY 4.0 license or if the copyright holder’s requirements are incompatible with the CC BY 4.0 license, please either i) remove the figure or ii) supply a replacement figure that complies with the CC BY 4.0 license. Please check copyright information on all replacement figures and update the figure caption with source information. If applicable, please specify in the figure caption text when a figure is similar but not identical to the original image and is therefore for illustrative purposes only.

7. Please include captions for your Supporting Information files at the end of your manuscript, and update any in-text citations to match accordingly. Please see our Supporting Information guidelines for more information: http://journals.plos.org/plosone/s/supporting-information.

8. If the reviewer comments include a recommendation to cite specific previously published works, please review and evaluate these publications to determine whether they are relevant and should be cited. There is no requirement to cite these works unless the editor has indicated otherwise.

Additional Editor Comments :

Dear Authors,

Thank you for submitting your manuscript to our journal. Your study addresses an interesting and potentially important topic. However, after careful evaluation, we believe that the manuscript requires major revision before it can be considered further.

Several issues need to be addressed to improve the quality and clarity of the work.

We encourage you to revise the manuscript substantially and resubmit a version that more clearly supports its conclusions.

[Note: HTML markup is below. Please do not edit.]

Reviewers' comments:

Reviewer's Responses to Questions

**Comments to the Author**

1. Is the manuscript technically sound, and do the data support the conclusions?

The manuscript must describe a technically sound piece of scientific research with data that supports the conclusions. Experiments must have been conducted rigorously, with appropriate controls, replication, and sample sizes. The conclusions must be drawn appropriately based on the data presented.

Reviewer #1: Yes

**********

2. Has the statistical analysis been performed appropriately and rigorously?

Reviewer #1: Yes

**********

3. Have the authors made all data underlying the findings in their manuscript fully available?

The PLOS Data policy requires authors to make all data underlying the findings described in their manuscript fully available without restriction, with rare exception (please refer to the Data Availability Statement in the manuscript PDF file). The data should be provided as part of the manuscript or its supporting information, or deposited to a public repository. For example, in addition to summary statistics, the data points behind means, medians and variance measures should be available. If there are restrictions on publicly sharing data—e.g. participant privacy or use of data from a third party—those must be specified.

Reviewer #1: Yes

**********

4. Is the manuscript presented in an intelligible fashion and written in standard English?

PLOS ONE does not copyedit accepted manuscripts, so the language in submitted articles must be clear, correct, and unambiguous. Any typographical or grammatical errors should be corrected at revision, so please note any specific errors here.

Reviewer #1: Yes

**********

5. Review Comments to the Author

Please use the space provided to explain your answers to the questions above. You may also include additional comments for the author, including concerns about dual publication, research ethics, or publication ethics. (Please upload your review as an attachment if it exceeds 20,000 characters)

Reviewer #1: The authors present an FDG-PET–based machine learning framework using ADNI data, combining image-derived and region-based SUVr-derived features from the same scan for classification of cognitively normal versus cognitively impaired/AD subjects. The manuscript is generally well organized, and the comparative evaluation of baseline, fusion, and ensemble models is a strength. The study suggests that combining these two FDG-PET–derived representations may improve classification performance within this dataset.

However, I have several major concerns. The manuscript often frames FDG-PET in a way that appears to overextend general advantages of PET or molecular imaging that do not always apply specifically to FDG-PET, particularly with respect to molecular specificity and early detection of AD. In addition, the novelty is somewhat overstated, since both feature sets are derived from the same underlying FDG-PET scan rather than from distinct modalities or independent biological sources. I am also concerned by the repeated use of terms such as “quantification,” when the described analysis appears to involve regional SUVr extraction rather than quantitative PET analysis. Overall, the main findings are best interpreted as improved comparative model performance within an internal ADNI-based ML framework, and I encourage the authors to revise the framing and terminology throughout the manuscript accordingly.

A few comments and concerns as follows:

Major 1. Page 4, lines 83-85. The statement that FDG-PET plays a key role in the early detection of AD seems factually imprecise and potentially misleading. FDG-PET is better considered a marker of downstream neurodegenerative/metabolic change, whereas amyloid and tau biomarkers are more directly tied to early AD pathology.

More broadly, the manuscript appears to repeatedly attribute to FDG-PET some of the molecular specificity advantages that are more characteristic of other PET tracers or biomarker frameworks. The authors should carefully re-examine the wording throughout the manuscript and revise statements that overgeneralize PET’s molecular specificity in a way that does not accurately apply to FDG-PET.

Major 2. Page 5, lines 108-112. The logic in this paragraph is unclear. The authors first highlight inter-institutional variability in scanner resolution and imaging protocols as a key limitation to generalizability, but then shift to the need for models that perform well on small datasets. These are not equivalent issues. Good performance in small datasets does not by itself address cross-scanner, cross-site, or protocol-related heterogeneity. The authors should clarify this rationale and avoid implying that limited-data modeling alone can overcome institutional variability in PET acquisition.

Major 3. Page 9, lines 180-185. The term “quantification” appears imprecise here. As described, the analysis involves extraction/calculation of regional SUVr values rather than quantitative PET modeling. This terminology may overstate the nature of the PET analysis and should be revised accordingly. Because similar wording appears elsewhere in the manuscript, the authors should review and correct this terminology throughout the paper for greater methodological precision.

Major 4. Page 18, lines 367-370. The wording here is somewhat vague and may overstate the methodological novelty. As described in the Methods, both the image-derived and region-based features are derived from the same FDG-PET data, with the latter representing ROI-level SUVr values. Thus, the framework appears to combine two representations of the same modality rather than fundamentally different data sources. The authors should clarify this point and describe more explicitly how these complementary representations provide additional information.

Major 5. Page 20, Conclusion. The Conclusion appears somewhat overstated relative to the actual scope of the study. The reported results mainly show improved internal classification performance within ADNI when combining two FDG-PET-derived representations, rather than establishing early detection capability, clinical relevance, or superior disease differentiability in a broader diagnostic sense. In particular, the statements regarding “early detection of Alzheimer’s disease,” MMSE correlation “confirming” clinical relevance, and better disease differentiability compared with MMSE should be toned down. The conclusion would be more appropriate if it emphasized improved comparative model performance within this dataset and framed the clinical implications more cautiously.

Minor 1. Page 19, lines 397-399. This statement is unclear. The need for segmentation and region-based SUVR extraction does not by itself explain why only a small dataset was available, nor why such a dataset would better reflect real-world clinical settings. Please clarify the reason for the reduced sample size.

**********

6. PLOS authors have the option to publish the peer review history of their article (what does this mean?). If published, this will include your full peer review and any attached files.

If you choose “no”, your identity will remain anonymous but your review may still be made public.

**Do you want your identity to be public for this peer review?**  For information about this choice, including consent withdrawal, please see our Privacy Policy.

Reviewer #1: No

**********

[NOTE: If reviewer comments were submitted as an attachment file, they will be attached to this email and accessible via the submission site. Please log into your account, locate the manuscript record, and check for the action link "View Attachments". If this link does not appear, there are no attachment files.]

To ensure your figures meet our technical requirements, please review our figure guidelines: https://journals.plos.org/plosone/s/figures

You may also use PLOS’s free figure tool, NAAS, to help you prepare publication quality figures: https://journals.plos.org/plosone/s/figures#loc-tools-for-figure-preparation.

NAAS will assess whether your figures meet our technical requirements by comparing each figure against our figure specifications.

# Author comment: Author response to Decision Letter 1

**DOI:** 10.1371/journal.pone.0341995.r002

20 Jun 2026

June 17, 2026

Dear Editor,

We would like to thank the reviewers for their insightful comments on our manuscript titled “Feature Integration of [18F]FDG PET Brain Imaging Using Deep Learning for Sensitive Cognitive Decline Detection” (PONE-D-26-02360). We have carefully considered each point raised and made the necessary revisions to the manuscript. Below, we provide a detailed response to each comment.

Editor Comments

Comment 1: Manuscript formatting and PLOS ONE style requirements

Response: We have revised the manuscript formatting in accordance with PLOS ONE style requirements, including Supporting Information file naming, title formatting, citations, and the symbol notation used for the consortium.

Comment 2: Code sharing policy

Response: We thank the reviewer for this suggestion. In accordance with PLOS ONE’s code sharing policy, we have deposited the author-generated code in a publicly accessible GitHub repository to facilitate reproducibility and reuse.

Repository: https://github.com/leeyoujin0221/feature_integration_AD

We have revised the Data Availability Statement accordingly and included the repository link in the manuscript.

Comment 3 and 5: ADNI consortium author information

Response: Thank you for this comment. The Alzheimer’s Disease Neuroimaging Initiative (ADNI) includes a very large number of contributors across multiple institutions. Following common practice in ADNI-based publications, we have acknowledged the consortium as a group author and provided a reference to the official ADNI Acknowledgement List rather than listing the individual authors in the manuscript. This approach has also been adopted in recent ADNI-based publications, including Gonzales et al. (2026), “White matter microstructure disruption associated with PET and cognitive impairment in Alzheimer’s disease” (PLOS ONE, 21(4): e0346661; https://doi.org/10.1371/journal.pone.0346661).

Accordingly, we have revised the Acknowledgments section to include the official ADNI Acknowledgement List (https://adni.loni.usc.edu/wp-content/uploads/how_to_apply/ADNI_Acknowledgement_List.pdf).

Comment 4: Funding statement correction

Response: We removed funding-related text from the manuscript in accordance with PLOS ONE policy and provided the corrected Funding Statement in the cover letter for updating the online submission record.

Comment 6: Figure 1 copyright compliance

Response: We contacted ADNI regarding copyright permission for the representative ADNI-derived image included in Figure 1 and obtained written permission for publication under the CC BY 4.0 license. The signed permission form has been provided as supporting documentation.

Comment 8: Reviewer-suggested references

Response: Thank you for this comment. We reviewed the reviewer comments and confirmed that no specific recommendations for additional citations were provided.

Reviewer 1

Thank you for your comments. We have revised the manuscript to improve its clarity and readability. We also updated the reported improvement percentage in the Abstract by recalculating it using the original (unrounded) values rather than the rounded values shown in the table; this update does not affect the reported performance values or the conclusions of the study. Each comment is addressed in detail below.

Major 1. Page 4, lines 83-85. The statement that FDG-PET plays a key role in the early detection of AD seems factually imprecise and potentially misleading. FDG-PET is better considered a marker of downstream neurodegenerative/metabolic change, whereas amyloid and tau biomarkers are more directly tied to early AD pathology.

More broadly, the manuscript appears to repeatedly attribute to FDG-PET some of the molecular specificity advantages that are more characteristic of other PET tracers or biomarker frameworks. The authors should carefully re-examine the wording throughout the manuscript and revise statements that overgeneralize PET’s molecular specificity in a way that does not accurately apply to FDG-PET.

Response: We thank the reviewer for this important comment. We agree that FDG-PET primarily reflects downstream neurodegenerative and metabolic changes rather than early AD-specific molecular pathology. Accordingly, we carefully reviewed and revised the relevant statements throughout the manuscript to avoid overstating the molecular specificity or early detection role of FDG-PET.

Specifically, the statement has been revised as follows (Page 4, lines 80-82): “Among current diagnostic tools, positron emission tomography (PET) plays an important role in the evaluation of AD, with different tracers providing complementary diagnostic information [17-19].” The corresponding references have also been updated accordingly.

Major 2. Page 5, lines 108-112. The logic in this paragraph is unclear. The authors first highlight inter-institutional variability in scanner resolution and imaging protocols as a key limitation to generalizability, but then shift to the need for models that perform well on small datasets. These are not equivalent issues. Good performance in small datasets does not by itself address cross-scanner, cross-site, or protocol-related heterogeneity. The authors should clarify this rationale and avoid implying that limited-data modeling alone can overcome institutional variability in PET acquisition.

Response: We thank the reviewer for this helpful comment. We agree that our original wording incorrectly linked inter-institutional acquisition variability with limited-data model performance, which are distinct challenges. Our intended point was that PET imaging is costly and requires radioactive tracers, which restrict large-scale data acquisition and result in relatively limited datasets for model development. We have revised the paragraph accordingly to clarify this rationale.

Specifically, the statement has been revised as follows (Page 5, lines 106-110): “However, PET imaging requires radioactive tracers and dedicated imaging infrastructure, making data acquisition expensive and comparatively limited relative to other imaging modalities [33, 34]. Consequently, developing models that can achieve robust performance with limited training data may enhance the clinical utility of PET-based diagnostic tools.” The corresponding references have also been updated accordingly.

Major 3. Page 9, lines 180-185. The term “quantification” appears imprecise here. As described, the analysis involves extraction/calculation of regional SUVr values rather than quantitative PET modeling. This terminology may overstate the nature of the PET analysis and should be revised accordingly. Because similar wording appears elsewhere in the manuscript, the authors should review and correct this terminology throughout the paper for greater methodological precision.

Response: We thank the reviewer for this important comment. We agree that the term “quantification” may overstate the nature of our PET analysis, as our approach involved extraction of regional PET measurements (SUVr values) rather than formal quantitative PET modeling. Accordingly, we revised the terminology throughout the manuscript for greater methodological precision, replacing “quantification” with context-appropriate terms such as “region-level PET measurements” and “regional SUVr values,” and clarifying that SUVr represents a semi-quantitative PET measure.

Specifically, the statement has been revised as follows (Page 9, lines 178-183): “Another limitation is the relatively small dataset, which reflects the practical constraints of PET-based research, where data availability is often limited due to acquisition cost and complexity [33, 34]. Preprocessing steps such as MRI segmentation and regional SUVr extraction also introduce substantial workflow burden, which may limit scalability in broader clinical setting. Despite of these limitations, evaluating models under such data-constrained conditions remains practically important, as these constraints are commonly encountered in real-world clinical practice.”

Major 4. Page 18, lines 367-370. The wording here is somewhat vague and may overstate the methodological novelty. As described in the Methods, both the image-derived and region-based features are derived from the same FDG-PET data, with the latter representing ROI-level SUVr values. Thus, the framework appears to combine two representations of the same modality rather than fundamentally different data sources. The authors should clarify this point and describe more explicitly how these complementary representations provide additional information.

Response: We thank the reviewer for this insightful comment. We agree that our framework integrates complementary representations derived from the same FDG-PET modality rather than fundamentally different data sources. Our intention was to emphasize the complementary nature of multi-scale representations rather than multimodal integration. We have revised the text to clarify that voxel-level image features capture distributed spatial metabolic patterns, whereas region-level PET measurements provide anatomically summarized information that may offer complementary discriminatory value.

Specifically, the statement has been revised as follows (Page 18, lines 364-369): “In our study, we developed a deep learning framework that leverages complementary representation derived from the same FDG-PET modality by integrating voxel-level image features with region-level PET measurements. While voxel-level representations capture spatially distributed metabolic patterns, region-level measurements provide anatomically aggregated information that may improve robustness and interpretability.”

Major 5. Page 20, Conclusion. The Conclusion appears somewhat overstated relative to the actual scope of the study. The reported results mainly show improved internal classification performance within ADNI when combining two FDG-PET-derived representations, rather than establishing early detection capability, clinical relevance, or superior disease differentiability in a broader diagnostic sense. In particular, the statements regarding “early detection of Alzheimer’s disease,” MMSE correlation “confirming” clinical relevance, and better disease differentiability compared with MMSE should be toned down. The conclusion would be more appropriate if it emphasized improved comparative model performance within this dataset and framed the clinical implications more cautiously.

Response: We thank the reviewer for this thoughtful comment. We agree that several statements in the original conclusion overstated the scope of our findings. Our results demonstrate improved comparative classification performance within the ADNI dataset rather than establishing broader clinical diagnostic utility or early detection capability. Accordingly, we revised the conclusion to adopt a more cautious interpretation, clarifying that MMSE correlations indicate consistency with cognitive measures rather than confirming clinical relevance.

Specifically, conclusion has been revised as follows (Page 20): “This study presented a deep learning framework that integrates complementary voxel-level and region-level representations derived from FDG PET data for cognitive decline classification. The proposed fusion framework achieved improved performance over single-representation models on the ADNI dataset, particularly in identifying cognitively impaired individuals. A moderate correlation with MMSE scores suggests alignment between model outputs and established cognitive assessments. These findings support the potential utility of multi-scale FDG-PET representations in machine learning–based cognitive decline detection, with implications for clinical diagnostic aid.”

Minor 1. Page 19, lines 397-399. This statement is unclear. The need for segmentation and region-based SUVR extraction does not by itself explain why only a small dataset was available, nor why such a dataset would better reflect real-world clinical settings. Please clarify the reason for the reduced sample size.

Response: We thank the reviewer for this helpful comment. We agree that our original wording did not clearly explain the rationale for the limited dataset size. Our intention was not to suggest that segmentation or SUVr extraction directly determined sample availability, but rather that PET-based studies often face practical data constraints due to the high cost of PET imaging, the requirement for radioactive tracers, and the time-intensive preprocessing workflow (e.g., MRI segmentation and regional SUVr extraction). We have revised the text accordingly to clarify this point.

Specifically, the statement has been revised as follows (Page 19, lines 397-404): “Another limitation is the relatively small dataset, which reflects the practical constraints of PET-based research, where data availability is often limited due to acquisition cost and complexity [33, 34]. Preprocessing steps such as MRI segmentation and regional SUVr extraction also introduce substantial workflow burden, which may limit scalability in broader clinical setting. Despite of these limitations, evaluating models under such data-constrained conditions remains practically important, as these constraints are commonly encountered in real-world clinical practice.”

Attachment Submitted filename: Response to Reviewers.docx

# Peer review: Decision Letter 1

**DOI:** 10.1371/journal.pone.0341995.r003

**Authors:** Emanuele Bartolini

1 Jul 2026

Feature Integration of [18F]FDG PET Brain Imaging Using Deep Learning for Sensitive Cognitive Decline Detection

PONE-D-26-02360R1

Dear Dr. Kang,

We’re pleased to inform you that your manuscript has been judged scientifically suitable for publication and will be formally accepted for publication once it meets all outstanding technical requirements.

Within one week, you’ll receive an e-mail detailing the required amendments. When these have been addressed, you’ll receive a formal acceptance letter and your manuscript will be scheduled for publication.

An invoice will be generated when your article is formally accepted. Please note, if your institution has a publishing partnership with PLOS and your article meets the relevant criteria, all or part of your publication costs will be covered. Please make sure your user information is up-to-date by logging into Editorial Manager at Editorial Manager® and clicking the ‘Update My Information' link at the top of the page. For questions related to billing, please contact billing support.

If your institution or institutions have a press office, please notify them about your upcoming paper to help maximize its impact. If they’ll be preparing press materials, please inform our press team as soon as possible -- no later than 48 hours after receiving the formal acceptance. Your manuscript will remain under strict press embargo until 2 pm Eastern Time on the date of publication. For more information, please contact onepress@plos.org.

Kind regards,

Emanuele Bartolini, MD

Academic Editor

PLOS One

Additional Editor Comments (optional):

Dear Authors,

your revised manuscript has now filled the original defects and is suitable for publication.

Reviewers' comments:

Reviewer's Responses to Questions

**Comments to the Author**

1. If the authors have adequately addressed your comments raised in a previous round of review and you feel that this manuscript is now acceptable for publication, you may indicate that here to bypass the “Comments to the Author” section, enter your conflict of interest statement in the “Confidential to Editor” section, and submit your "Accept" recommendation.

Reviewer #1: All comments have been addressed

**********

2. Is the manuscript technically sound, and do the data support the conclusions?

The manuscript must describe a technically sound piece of scientific research with data that supports the conclusions. Experiments must have been conducted rigorously, with appropriate controls, replication, and sample sizes. The conclusions must be drawn appropriately based on the data presented.

Reviewer #1: Yes

**********

3. Has the statistical analysis been performed appropriately and rigorously?

Reviewer #1: Yes

**********

4. Have the authors made all data underlying the findings in their manuscript fully available?

The PLOS Data policy requires authors to make all data underlying the findings described in their manuscript fully available without restriction, with rare exception (please refer to the Data Availability Statement in the manuscript PDF file). The data should be provided as part of the manuscript or its supporting information, or deposited to a public repository. For example, in addition to summary statistics, the data points behind means, medians and variance measures should be available. If there are restrictions on publicly sharing data—e.g. participant privacy or use of data from a third party—those must be specified.

Reviewer #1: Yes

**********

5. Is the manuscript presented in an intelligible fashion and written in standard English?

PLOS ONE does not copyedit accepted manuscripts, so the language in submitted articles must be clear, correct, and unambiguous. Any typographical or grammatical errors should be corrected at revision, so please note any specific errors here.

Reviewer #1: Yes

**********

6. Review Comments to the Author

Please use the space provided to explain your answers to the questions above. You may also include additional comments for the author, including concerns about dual publication, research ethics, or publication ethics. (Please upload your review as an attachment if it exceeds 20,000 characters)

Reviewer #1: The authors have adequately addressed my previous comments. The revised manuscript now presents the FDG-PET framing and SUVr-based methodology more accurately, and the main claims are better aligned with the presented results. I have no further major concerns.

**********

7. PLOS authors have the option to publish the peer review history of their article (what does this mean?). If published, this will include your full peer review and any attached files.

If you choose “no”, your identity will remain anonymous but your review may still be made public.

**Do you want your identity to be public for this peer review?**  For information about this choice, including consent withdrawal, please see our Privacy Policy.

Reviewer #1: No

**********

# Editor report: Acceptance letter

**DOI:** 10.1371/journal.pone.0341995.r004

**Authors:** Emanuele Bartolini

PONE-D-26-02360R1

PLOS One

Dear Dr. Kang,

I'm pleased to inform you that your manuscript has been deemed suitable for publication in PLOS One. Congratulations! Your manuscript is now being handed over to our production team.

At this stage, our production department will prepare your paper for publication. This includes ensuring the following:

* All references, tables, and figures are properly cited

* All relevant supporting information is included in the manuscript submission,

* There are no issues that prevent the paper from being properly typeset

You will receive further instructions from the production team, including instructions on how to review your proof when it is ready. Please keep in mind that we are working through a large volume of accepted articles, so please give us a few days to review your paper and let you know the next and final steps.

Lastly, if your institution or institutions have a press office, please let them know about your upcoming paper now to help maximize its impact. If they'll be preparing press materials, please inform our press team within the next 48 hours. Your manuscript will remain under strict press embargo until 2 pm Eastern Time on the date of publication. For more information, please contact onepress@plos.org.

You will receive an invoice from PLOS for your publication fee after your manuscript has reached the completed accept phase. If you receive an email requesting payment before acceptance or for any other service, this may be a phishing scheme. Learn how to identify phishing emails and protect your accounts at https://explore.plos.org/phishing.

If we can help with anything else, please email us at customercare@plos.org.

Thank you for submitting your work to PLOS ONE and supporting open access.

Kind regards,

PLOS ONE Editorial Office Staff

on behalf of

Dr. Emanuele Bartolini

Academic Editor

PLOS One
